# Low-dose oral minoxidil for persistent chemotherapy and radiotherapy-induced alopecia in a pediatric female patient^⋆^

**DOI:** 10.1016/j.abd.2023.07.010

**Published:** 2024-04-23

**Authors:** Raíssa Rodriguez Melo, Rita Fernanda Cortez de Almeida, Luciana Rodino Lemes, Sidney Frattini Junior, Paulo Müller Ramos, Daniel Fernandes Melo

**Affiliations:** a Dermatology Department, Universidade do Estado do Rio de Janeiro, Rio de Janeiro, RJ, Brazil; b The Mole Clinic, Private Practice, Ancaster, Ontario, Canada; c Department of Infectious Diseases, Dermatology, Imaging Diagnosis and Radiotherapy, Faculty of Medicine, Universidade Estadual Paulista, Botucatu, SP, Brazil; d Dermatology Department, Universidade do Estado do Rio de Janeiro (UERJ), Rio de Janeiro, RJ, Brazil

Dear Editor,

Cytotoxic chemotherapy, molecularly targeted therapy, immunotherapy, radiotherapy, stem cell transplant, and endocrine therapies may lead to hair disorders which in most cases are reversible.^1^, ^2^ However, persistent Chemotherapy-Induced Alopecia (pCIA) and persistent Radiotherapy-Induced Alopecia (pRIA) can occur.^1^, ^2^ They are defined as incomplete hair regrowth more than 6 months after treatment conclusion.^2^ It depends on the type, duration, and dose of oncological treatment.^1^ This article reports a pediatric female patient with pCIA and pRIA, who showed significant hair regrowth using Low-Dose Oral Minoxidil (LDOM).

A 4-year-old female patient was diagnosed with Atypical Rhabdoid Teratoid Tumor (ARTT) of the cerebellum. Dana-Farber protocol chemotherapy was initiated with vincristine, cisplatin, doxorubicin, cyclophosphamide, etoposide, temozolomide, and actinomycin D for 18 months. In addition, she was submitted to 10 sessions of intrathecal chemotherapy with cytarabine and dexamethasone, followed by skull and neuraxis radiotherapy, and an occipital craniotomy with partial tumor resection. Fortunately, she achieved oncological remission but evolved with pCIA and pRIA. At age 9, alopecia became a cosmetic concern, so 5% topical minoxidil was tried once daily for 6-months with no clinical response. At age 14, LDOM was started at 0.5 mg/day with some improvement in hair density after 6 months. This dose was increased to 1 mg/day for another 6 months with remarkable clinical and trichoscopic response and no reported side effects (Figure 1, Figure 2).

**Figure 1:**
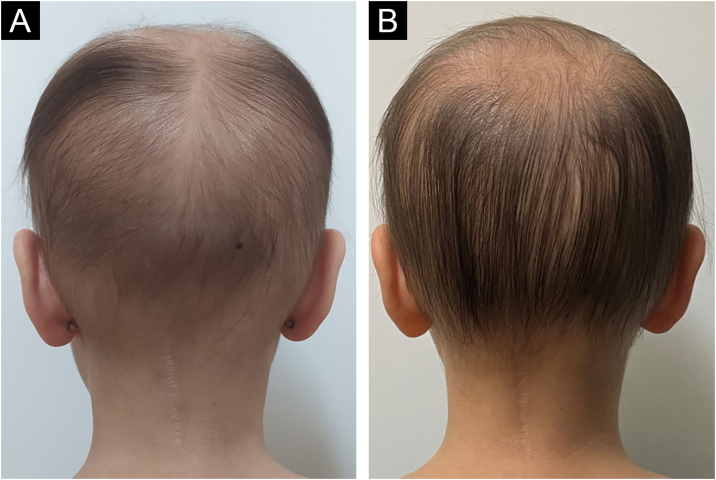
(A) Clinical photograph of female pediatric patient with pRIA and pCIA before treatment, showing diffuse alopecia with thin hairs. (B) Clinical image of the same patient after one year of treatment with LDOM showing improvement of hair density.

**Figure 2:**
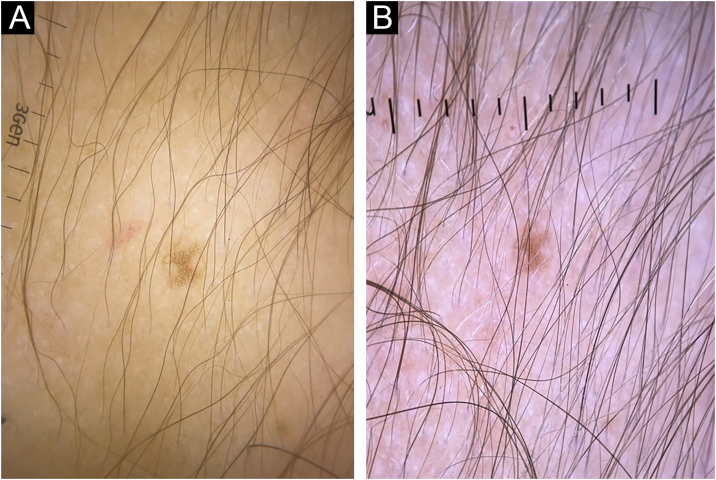
(A) Trichoscopy image low hair density with a predominance of thin hairs before treatment with LDOM. (B) Trichoscopy image of the same area, showing a significant improvement in hair density and predominance of thicker hair shafts after one year of LDOM treatment.

pCIA has been reported in 14% of childhood cancer survivors.^1^ Acute hair loss during chemotherapy occurs due to the cytotoxic action on the hair follicle, interrupting mitosis and disturbing the hair cycle. The exact mechanisms that lead to pCIA and pRIA are unclear but may be related to damage to follicle stem cells and altered signaling with failure to restore a new cycle.^2^, ^3^ The association of chemotherapy and radiotherapy increases the risks of persistent alopecia.^2^

Busulfan, cyclophosphamide, anthracycline, carboplatin, docetaxel, paclitaxel, and etoposide are the agents most commonly associated with pCIA. Clinically, pCIA may present similar to androgenetic alopecia, with a diffuse or patchy pattern or total alopecia. Histopathology often shows non-scarring alopecia with reduced hair density and miniaturization.^3^

Oral minoxidil is an arterial vasodilator first introduced as an antihypertensive medication.^4^, ^5^ In dermatology, LDOM (0.25‒5 mg/day) has been increasingly used for androgenetic alopecia, alopecia areata, traction alopecia, and, more recently, pCIA.^4^, ^5^ For pRIA in the pediatric population, LDOM has not yet been reported. A retrospective study with 63 pediatric patients treated with LDOM for different types of hair loss showed no serious adverse effects.^5^

pCIA and pRIA can cause significant distress, impacting the psychosocial development of children and adolescents. It is particularly challenging when they co-occur. We presented a case of associated pCIA and pRIA successfully treated with LDOM. Despite the few reports in the literature of this medication in the pediatric age group for persistent hair loss conditions, it seems to be safe and effective and should be considered by dermatologists.

## Financial support

None declared.

## Authors’ contributions

Raíssa Rodriguez Melo: Prepared the draft; wrote the manuscript and approved the final version to be published.

Rita Fernanda Cortez de Almeida: Designed the study; wrote the manuscript and approved the final version to be published.

Luciana Rodino Lemes: Designed the study; wrote the manuscript and approved the final version to be published.

Sidney Frattini Junior: prepared the draft, reviewed the text and approved the final version to be published.

Paulo Muller Ramos: Designed the study; critically reviewed the manuscript and approved the final version to be published.

Daniel Fernandes Melo: Conceived the study; critically reviewed the manuscript and approved the final version to be published.

## Conflicts of interest

None declared.
